# Fitness bloggers as health information suppliers: how content production ability and reputation serve as quality signals and shape knowledge dissemination in Chinese online communities

**DOI:** 10.3389/fdgth.2026.1873602

**Published:** 2026-08-04

**Authors:** Chaoran Li, Luxi Xu

**Affiliations:** School of Economics and Management, Shanghai University of Sport, Shanghai, China

**Keywords:** fitness knowledge, health information dissemination, information asymmetry, internet fitness, regression analysis, signaling theory

## Abstract

**Introduction:**

The proliferation of Internet fitness has fostered online fitness communities (OFCs), where fitness knowledge is produced, shared, and consumed as a health-related good. In this digital health information market, information asymmetry between fitness bloggers (suppliers) and users (consumers) necessitates reliance on observable quality signals. Drawing on signaling theory, this study examines how content production ability and reputation influence the spread breadth and spread recognition of fitness knowledge.

**Methods:**

Using real-world behavioral data from 1,561 fitness bloggers on a popular Chinese OFC, hierarchical OLS regression models were constructed to test the proposed hypotheses. Spread breadth and spread recognition were measured by one-month increments in video views and likes, respectively.

**Results:**

Content production ability positively affected both spread breadth and spread recognition, whereas reputation only increased spread breadth. Additionally, the effect of content production ability on both spread dimensions was heterogeneous across genders, while male fitness bloggers benefiting more.

**Discussion:**

These findings contribute to the understanding of supply-side signal mechanisms in health information markets and offer practical insights for optimizing resource allocation in digital health promotion.

## 1 Introduction

With the flourishing of the Internet and the increasing use of social media, our daily lives are undergoing a substantial digital transformation. In the realm of sport, our engagement with physical activities and healthy living has been changed by the advent of the Internet (1, 2); the way people acquire fitness knowledge is changing. The online fitness community (OFC) has become an important part of Internet fitness. OFC mainly focuses on fitness and sports. As one kind of online virtual community, OFC provides an online environment for fitness bloggers to share and spread fitness knowledge. At the same time, fitness enthusiasts can not only easily obtain professional fitness knowledge and personalized training experience generated by fitness bloggers but also communicate with each other (including fitness bloggers and other users) to share their views and experiences. Online fitness services enable people to overcome traditional fitness clubs' temporal and spatial limitations. People can now work out at their convenience, following training videos online. A body of research points to the efficacy of online fitness services in fostering increased physical activity levels (1, 3).

Social media enables individuals to participate in the “wider online community” (4), and tremendously facilitates and accelerates the creation, consumption, and spread of information (5). More and more people are using the internet and social media to share personal fitness experience and access others' shared experiences and information (6), especially, based on Facebook, Twitter, TikTok and other social media, which helps spread fitness information (7). OFC also functions as social media; the popularity and broad application of OFC have further accelerated the spread and sharing of fitness knowledge, and help to achieve “sports for all”.

Existing studies on Internet fitness or online fitness mainly fall into two categories. The first category, using qualitative research methods, mainly focused on the Internet fitness's social and economic impact, such as public fitness behavior. Jong et al. (8) highlighted that online fitness has gained traction as a recreational choice and a reliable channel for fitnesss-related information. Compared with traditional fitness methods, online fitness courses have many advantages like convenience, accessibility, low cost, and community interaction. However, they often suffer from high abandonment rates and low adherence (9, 10) used thematic analysis to explore the perceptions of people with multiple sclerosis regarding social interaction, technological accessibility, and sense of community in online fitness. They suggested that online fitness enhances flexibility and safety in exercise, but faces challenges such as technical barriers, insufficient personalization, and limited social interaction. The second category, using quantitative research methods such as the structural equation model, mainly focused on the influence of Internet fitness on individual behavior. Liu et al. (11) found that fitness applications and online fitness live-streaming courses significantly encourage users to engage in physical exercise, helping them reach the recommended amount of physical activity. Several researchers have extensively investigated the motivational mechanisms of fitness video, emphasizing that the credibility and appeal of fitness influencers significantly increase users' physical activity (12, 13). In addition, Liu et al. (14) found that fitness influencers' content quality also positively impacts the audience's willingness to exercise. However, there are limited studies focused on the fitness knowledge creators (e.g., online fitness bloggers) and the spread of fitness knowledge they generated on the Internet. In Internet fitness, online fitness bloggers are the crucial part of developing the Internet fitness ecology. The fitness knowledge they generate will be spread to various places on the Internet through online fitness platforms or social media, so that ordinary users and fitness enthusiasts can obtain a lot of fitness knowledge. The utilization of social media platforms has significantly facilitated the evolution and dissemination of sports-related content, necessitating the adoption of computational methodologies in social media analytics to investigate emerging trends and contextual dynamics within the sports domain (15).

From a health economics standpoint, the online distribution of fitness knowledge can be conceptualized as a market where health-related information is produced, exchanged, and consumed. In this market, fitness bloggers act as suppliers of a good—fitness knowledge—that has direct implications for health behavior and population-level public health outcomes. However, digital health information markets are characterized by high information asymmetry: consumers often lack the expertise to accurately evaluate the credibility and quality of health content on social media (16). In such conditions, the observable attributes of suppliers—such as their content production ability and reputation—may function as quality signals that reduce uncertainty and influence the efficiency of knowledge diffusion. Recent systematic reviews have applied signaling theory to examine how quality signals operate amidst information asymmetry in online health communities (17). Systematic evidence also demonstrates that digital health interventions can yield favorable cost-effectiveness outcomes (18) and that consumers exhibit measurable willingness to pay for eHealth services (19), collectively underscoring the practical importance of efficient digital health information dissemination. Understanding how these supply-side characteristics shape the breadth and depth of information spread is therefore critical for optimizing the allocation of health promotion resources and for designing cost-effective digital interventions aimed at improving physical activity levels. Against this backdrop, examining fitness bloggers' features through a health economics lens offers a novel perspective on the mechanisms driving the propagation of health-enhancing information.

Drawing on a health economics perspective and addressing these gaps, this research investigates the spread of fitness knowledge created by fitness bloggers in online fitness communities (OFCs), aiming to explore how fitness bloggers' features influence the spread effect of fitness knowledge on the Internet. This research summarized the features of online fitness bloggers into two aspects: content production ability and reputation. In addition, this study regarded gender as a moderating variable to explore whether there is heterogeneity between different groups of fitness bloggers. This research aims to answer the below questions:

1. How does the content production ability of fitness bloggers affect the spread of fitness knowledge?
2. How does the reputation of fitness bloggers affect the spread of fitness knowledge?
3. How does gender moderate the impact of fitness bloggers' features on the spread effect?

To address the aforementioned research questions, this research proposed a series of theoretically-grounded hypotheses derived from extant literatures. Employing a dataset comprising 1,561 fitness bloggers from Bilibili's Aerobics category, one of the popular online fitness communities in China, this research constructed an empirical model to test these hypotheses.

## 2 Literature review

### 2.1 Online fitness community

In this study, we regard an online fitness community as a kind of online virtual community in which the topic is sports, fitness, and other relevant areas. Within these platforms, users can upload the outcomes of their exercise sessions and various physical endeavors (20). Online fitness communities serve two fundamental purposes: (1) providing support for physical activity engagement, and (2) facilitating the exchange of social interactions and lifestyle experiences related to fitness.

Most scholars find that OFCs positively influence fitness activity by enabling personal progress tracking, interpersonal connection, and hedonic experience cultivation (20, 21), while also fostering stronger achievement motivation and peer interaction (22, 23). The growing prominence of OFCs has prompted scholarly investigation from diverse disciplinary perspectives and methods. Online fitness videos and live webcasts have attracted the attention of many scholars. Against the backdrop of growing health consciousness, rising demand for physical activity, and evolving exercise modalities, fitness videos have emerged as a dominant medium for health promotion and spread of fitness knowledge and techniques. Sui et al. (9) believe that online fitness videos have obvious advantages, but the viewers' long-term participation and persistence ability are unknown. Thus, it is becoming increasingly important to find ways to improve user engagement and using online fitness videos. Some scholars have conducted in-depth research on online fitness live broadcasting, analyzed viewers' psychological and behavioral characteristics from different perspectives, and discussed how to enhance the attraction of live broadcasting through content innovation, technology application, and social interaction. Wang et al. (24) found that gamification elements can significantly improve the user activity and retention rate of fitness apps, and can effectively improve the user activity and retention rate. Livingston et al. (25) found that social power, primarily through positive emotions, can enhance individual health awareness and behavior. Live fitness platforms can use community effects to encourage user interaction and sharing, thus forming a positive community atmosphere and improving users' fitness motivation.

In addition, in the research field of fitness apps, the users' usage intention and continuous usage intention are the focus of the existing research. Yuan et al. (26) examined what factors impacting users to use fitness applications, and found that performance expectations, hedonic motivation, price, and habits significantly impacted users to use fitness applications. Drawing upon status quo bias theory and goal-setting theory, Li et al. (27) developed a moderated mediation framework to examine the determinants of users' intention to explore fitness applications (IEFA). Their investigation revealed that both perceived need and user inertia serve as significant antecedents of behavioral intention. However, limited literature explores the factors affecting the spread of fitness knowledge online, highlighting the need for in-depth research on this topic.

From a health economics perspective, fitness knowledge shared in OFCs can be conceptualized as a health-related good that is produced, distributed, and consumed in a digital market. Unlike physical goods, health information is an experience good whose quality is difficult to ascertain prior to consumption, giving rise to considerable information asymmetry between content suppliers (fitness bloggers) and consumers (users) (17). Indeed, health information consumers on social media often lack the requisite knowledge to evaluate content credibility (16). In such markets, observable blogger characteristics—such as content production capability and reputational cues—may function as quality indicators that reduce consumer uncertainty and shape the efficiency of knowledge diffusion. Despite the growing literature on OFCs, few studies have examined the spread of fitness knowledge through this health economics lens, particularly how supply-side signals influence both the reach and the recognition of health-promoting information. Addressing this gap is critical for understanding how to optimize the allocation of digital health information resources and enhance the cost-effectiveness of online health promotion strategies.

### 2.2 Spread of fitness knowledge

Most research on fitness knowledge is based mainly on psychology or sociology research methods. Employing questionnaire survey, some scholars explored the grasp of fitness knowledge of different groups such as students and children (28), and analyzed the relationship between individual fitness knowledge level and factors such as physical activity, physical fitness and cognition (29). Additionally, some scholars discussed the positive effect of fitness knowledge spread on the psychological cognition and behavior of the public. Silva-Jose et al. (30) examined the efficacy of online fitness interventions in enhancing physical activity engagement and psychological well-being among pregnant women during COVID-19 restrictions, subsequently proposing suggestions for optimizing digital prenatal exercise platforms.

Fitness bloggers have been shown to positively influence health behavior adoption (31), with factors such as knowledge consumption, social interaction, and trustworthiness enhancing users' exercise intentions (12, 32).

The above literature mainly focuses on the effect of fitness bloggers on public health behaviors, with limited attention to how fitness knowledge itself spreads and gains acceptance. In the information spread field, scholars have used spread breadth (information coverage) and spread recognition (audience acceptance and perceived value) to measure dissemination effectiveness (33, 34).

Therefore, in our study focused on the spread of fitness knowledge, we used spread breadth and spread recognition to measure the spread effect of fitness knowledge in online fitness communities. We also explored the factors that influence the spread effect of fitness knowledge. Research on the spread breadth and recognition of fitness knowledge can help better understand how fitness knowledge spreads on the Internet, and find what factors affect the different kinds of spread effects.

From a health economics standpoint, the efficiency with which fitness knowledge is disseminated directly influences the effectiveness of population-level health promotion and the return on digital health investments. Information that achieves broader spread at lower cost represents a more efficient allocation of scarce health communication resources. Recent systematic evidence confirms that social media influencers can impact health outcomes ranging from dietary behavior to vaccination uptake (35), underscoring the real-world significance of understanding how health information propagates through digital channels. However, existing studies have primarily focused on behavioral or psychological outcomes, leaving the supply-side determinants of dissemination efficiency—such as content production capacity and reputational mechanisms—largely unexplored. This study thus addresses this gap by investigating how fitness bloggers' observable characteristics influence the dual dimensions of dissemination efficiency: spread breadth (coverage) and spread recognition (acceptance), both of which are critical indicators of health information market performance.

## 3 Hypothesis development

**Figure 1:**
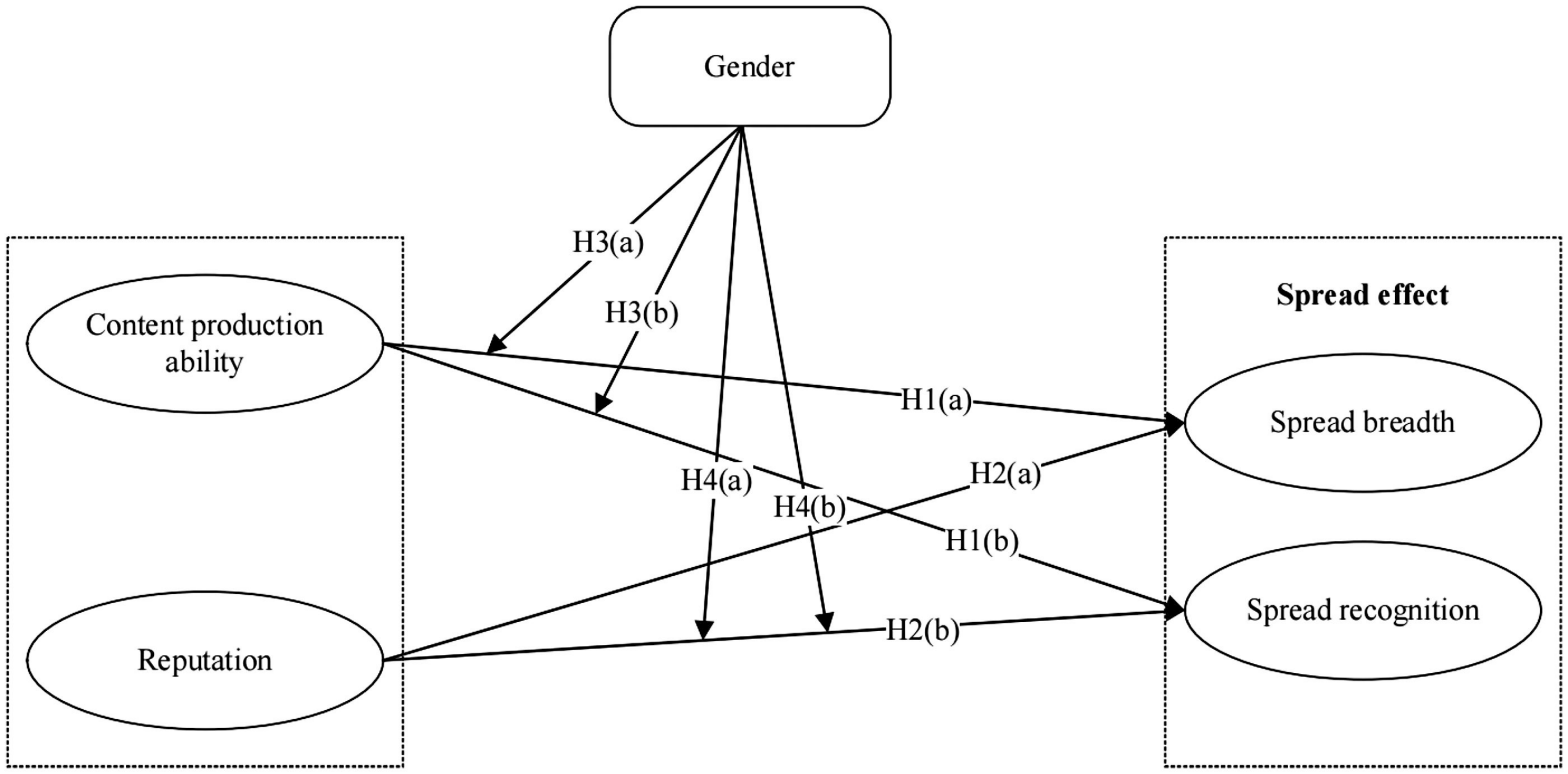
Research model.

This study aimed to explore how fitness bloggers' features influence the spread of fitness knowledge and to assess whether gender significantly moderates the relationship between these features and the spread effect. We used spread breadth and spread recognition as two measures to fully measure the spread effect of fitness knowledge on the Internet. This study put forward a series of hypotheses to reply the aforementioned research questions, and the proposed conceptual framework is shown in Figure 1.

In the context of a digital health information market characterized by information asymmetry, users must rely on observable signals to infer the quality and credibility of fitness knowledge before consumption. Drawing on signaling theory, this study conceptualizes fitness bloggers' content production ability and reputation as two distinct quality signals that may reduce consumer uncertainty and facilitate health knowledge transactions in OFCs. Content production ability signals supply-side capacity and commitment, indicating a blogger's capability to sustainably deliver health-related content. Reputation functions as a credibility signal derived from aggregated user feedback and social endorsement. This signaling framework allows us to derive theoretically grounded predictions about how each signal differentially influences the two dimensions of dissemination efficiency—spread breadth (the reach of health information) and spread recognition (the acceptance and perceived value of health information).

Ability is that people have certain skills and competencies in a specific domain and can provide high-quality products or services for users (36). Zhao et al. (36) demonstrated that answer frequency in paid Q&A platforms serves as a credible signal of knowledge contributor expertise and ability. The contributors' ability is an important reference for people to prejudge their answers' quality and positively impacts the users' payment decisions. Ahn et al. (37) identified ability as a crucial determinant for assessing sellers' service quality in e-commerce. Similarly, users are likelier to trust professional fitness bloggers who consistently and frequently deliver high-quality fitness content. The content production ability in this study can be understood as the ability of fitness bloggers to continuously and efficiently generate and share fitness-related knowledge posts through online platforms.

A fitness blogger's content production ability can be directly measured by the number of fitness blogs posted by a fitness blogger in a time period (38). Bloggers' online behavior determines their social roles and affects the way information spreads in online social networks. A blogger with high content production ability may quickly spread information to multiple people and influence them to spread information (39). Therefore, fitness bloggers with high content production ability can maintain a high frequency of content updates, which helps to improve the speed and breadth of fitness knowledge spread. From a health production perspective, higher content production ability reflects greater supplier efficiency in generating health information, which translates into more frequent exposure opportunities for users and may enhance both the volume and perceived value of health knowledge consumed. Thus, we proposed the following hypotheses:

H1(a): The content production ability of fitness bloggers has a positive influence on the spread breadth of their generated fitness knowledge in online communities.

H1(b): The content production ability of fitness bloggers has a positive influence on the spread recognition of their generated fitness knowledge in online communities.

Reputation refers to the popularity and word of mouth of an individual in the community (40, 41). Reputation manifests quantifiably via external feedback mechanisms (e.g., the number of followers) (42, 43) and functions as a critical determinant of users' attitudes (44). Since lacking direct experience, people must rely on secondary information like reputation to form their initial perception of their unfamiliar things. Reputation functions as a salient credibility indicator; existing research indicated that people perceived information as more useful if they perceived the source as having a higher reputation (45). Reputation facilitates rational decision-making by mitigating perceived risks and uncertainties in digital contexts (46), constituting a critical determinant of knowledge-sharing behaviors in online communities (47). Similarly, in the OFCs, due to the asymmetry of fitness information between fitness bloggers and users, users deficient in fitness-related knowledge exhibit greater dependence on observable information like reputation indicator during fitness blogger selection processes. In general, the more followers, the higher the blogger's reputation (41), the number of followers can be regarded as a measure of reputation. Highly reputable fitness influencers typically possess larger follower bases and expanded social networks, facilitating accelerated spread of fitness-related content posts to broader online audiences. In digital health contexts, reputation serves as an ex-ante credibility heuristic that lowers users’ perceived risk of adopting health advice, thereby potentially increasing information reach; however, its influence on deeper engagement such as recognition may be contingent on the actual consumption experience. Thus, we proposed the following hypotheses:

H2(a): The reputation of fitness bloggers has a positive influence on the spread breadth of their generated fitness knowledge in online communities.

H2(b): The reputation of fitness bloggers has a positive influence on the spread recognition of their generated fitness knowledge in online communities.

Gender constitutes a critical demographic variable for market segmentation strategies (48). Empirical evidence establishes that gender generates differential responses to identical stimuli (49). Consequently, gender frequently functions as a moderating variable across relational constructs. Sanchez-Franco et al. (50) specifically demonstrated its moderating influence on technology acceptance processes, while Dedeoglu (51) confirmed gender's moderation of relationships between information quality and both source trustworthiness perceptions and content-sharing importance. Additional studies further demonstrate gender's moderating effects within digital information contexts (52, 53).

Compared with female bloggers, male bloggers may differ in the impact of content production ability and reputation on the spread of fitness knowledge. The moderating effects of gender within the above established relationships remain empirically ambiguous, and need to be further investigated. Therefore, this study assumed that the influence of content production ability and reputation on the spread of fitness knowledge is heterogeneous between males and females. Thus, we propose the following hypotheses:

H3(a): The influence of fitness bloggers' content production ability on spread breadth is heterogeneous among different genders.

H3(b): The influence of fitness bloggers' content production ability on spread recognition is heterogeneous among different genders.

H4(a): The influence of fitness bloggers' reputation on spread breadth is heterogeneous among different genders.

H4(b): The influence of fitness bloggers' reputation on spread recognition is heterogeneous among different genders.

## 4 Research method

### 4.1 Research context

The Bilibili Aerobics Section (BAS; https://www.bilibili.com/v/sports/aerobics) was chosen as the research context for this research. Founded in 2010, Bilibili has evolved into one of the largest online entertainment communities in China, catering to a wide range of interests, including anime, gaming, and sports. The functions of Bilibili are similar to those of YouTube, where a user can create his or her channel and share, comment on, and interact with diverse content offerings. BAS is part of a broader sports category on Bilibili, featuring diverse fitness and aerobic-related content. Users can share content, including, but not limited to, training videos, exercise tips, coaching, and live tutorials. Everyone can freely access the contents shared within BAS.

Furthermore, BAS employs an authentication system for its fitness bloggers. These fitness bloggers can receive official badges, such as fitness champions, outstanding coaches, and BAS-certified content creators, on their channels after a reference check. BAS allows user engagement through various interactive features like bullet comments, likes, virtual gifts, and memberships. In China, BSA is one of the most popular OFCs, where people can obtain high-quality fitness knowledge and interact with each other to share fitness experiences. Many fitness bloggers are active on the platform and contribute all kinds of fitness knowledge. This OFC is also one of the important channels for spreading fitness knowledge and delivering fitness-for-all in China.

### 4.2 Data collection

The BAS website displays information about numerous fitness bloggers on its listing page. The platform employs its own display mechanism that researchers cannot alter, and the complete list of all bloggers cannot be accessed from the backend. Accordingly, we adopted a convenience sampling approach rather than a random sampling method. We manually visited the BAS website on multiple occasions, collected blogger homepage links, and compiled them into an initial list, eventually obtaining 2,000 blogger records.

We then developed a Python-based web crawler to systematically collect the publicly available homepage information of these 2,000 bloggers from April to June 2024. Following data collection, we conducted a comprehensive data cleaning procedure. Since the platform's display mechanism may present the same blogger across different visits, we identified 239 duplicate records and removed them, leaving 1,761 unique bloggers. We further excluded 170 bloggers who had not published fitness video content as of April 2024 and 30 bloggers whose accounts had been closed or whose data were incomplete. These records could not support variable extraction. After these cleaning procedures, the final empirical dataset comprised online information from 1,561 bloggers, including the number of followers, number of views, number of likes, number of posts, comments, personal bulletins, and gender.

### 4.3 Variable measurement

#### 4.3.1 Dependent variables

Refer to the research of Han et al. (33), Qiang et al. (54), and Gao et al. (34), this research categorized the spread effect into two dimensions: spread breadth and spread recognition. Spread breadth typically refers to the information coverage, the extent to which the information can reach various audience groups. On the display page of the BAS platform, every video shows the number of times it has been viewed. As the number of views increases, more viewers have been exposed to this video, which reflects that the information has been spread among a broader audience. Therefore, the spread breadth directly reflects the number of times a video has been viewed. To avoid reciprocal causation, this research used the increment of the view numbers in 1 month as a measurement indicator of spread breadth; the greater the increment in a given period, the greater the spread breadth achieved. We subtract the number of views as of April from those as of May to get the incremental number of views for 1 month.

Spread recognition reflects the degree of acceptance and recognition of the information content by the audience. Referring to the research of Gao et al. (34), likes indicate users' affirmation and appreciation of the content. Therefore, this research used the increment of the number of likes in 1 month as a measurement indicator of spread recognition; the greater the increment in a given period, the greater the spread recognition obtained. We subtract the number of likes as of April from those as of May to get the incremental number of likes for 1 month.

In general, the dependent variables in this study are the spread breadth and spread recognition of the fitness knowledge generated by the fitness bloggers in online communities. Both spread breath and spread recognition can describe the spread effect of fitness knowledge on the Internet more completely.

#### 4.3.2 Independent variables

In our research, independent variables are content production ability and reputation. Regarding content production ability, referring to the research of Khan et al. (38), it can be directly measured by the number of posts generated by the fitness bloggers over a period. The more fitness knowledge a blogger posts, the higher the fitness blogger's ability to produce fitness knowledge. So, this study uses the increments in the number of fitness knowledge videos posted by a blogger in 1 month to measure the fitness blogger's content production ability. We subtract the number of videos as of April from those as of May to get the incremental number of videos for 1 month.

In online communities, the number of followers of a blogger is displayed on his/her homepage. Existing studies showed that the number of followers was a signal to measure reputation (36). The number of follower indicates a blogger's community popularity and ability to attract audiences. Increased popularity and user loyalty collectively enhance the blogger's reputation (43). Therefore, a fitness blogger with more followers is considered to have more influence and a higher reputation. In this study, the number of followers can be regarded as an indicator to measure the reputation of a fitness blogger. We employ the number of followers as of April to measure the fitness blogger's reputation.

#### 4.3.3 Moderator variables and control variables

In this study, we regard gender as a moderator variable, and define a dummy variable, Gender, to show whether a fitness blogger is male. Gender = 1 if the blogger is male, but zero otherwise. Besides, age and socioeconomic background of a fitness blogger, while may also play as potential moderators, are not disclosed in publicly available blogger profiles on Bilibili and could not be included in this study.

In addition, we use the number of comments as of April (NumCom), the number of videos as of April (NumVideo), personal bulletin length (Length), online time (Time), and identity authentication (Auth) as control variables. These five control variables were selected to account for baseline differences in bloggers’ platform presence that may confound the estimated signaling effects. NumCom as a control variable to account for user engagement depth, which may influence dissemination outcomes independently of production ability and reputation. NumVideo controls for baseline content stock, as cumulative video volume mechanically affects 1-month dissemination increments. Length captures the blogger's self-presentation effort on their profile page, which credibly signals overall content investment to the audience (55). Time accounts for platform tenure effects, since longer-established bloggers benefit from accumulated algorithmic visibility and follower inertia (55). Auth controls for the trust premium conferred by official platform verification (56). Without these controls, the estimated effects of CPA and Rep on dissemination would be confounded by systematic baseline differences across bloggers.

The overview of dependent, independent, and control variables is shown in Table 1.

**Table 1:** Variables descriptions.

Variables	Description
Dependent variable	
Spread breadth (SB)	The increment in the number of views of fitness knowledge videos posted by a fitness blogger in 1 month.
Spread recognition (SR)	The increment in the number of likes that fitness knowledge videos of a fitness blogger received in 1 month.
Independent variables	
Content production ability (CPA)	The increment in the number of fitness knowledge videos posted by a fitness blogger in 1 month.
Reputation (Rep)	The total number of followers of a fitness blogger.
Moderator variable	
Gender	Dummy variable to show whether a fitness blogger is a male or female. Gender = 1 if male, but zero otherwise.
Control variables	
Number of comments (NumCom)	The total number of comments that a fitness blogger's fitness knowledge videos received from users as of April.
Number of videos (NumVideo)	The total number of fitness knowledge videos posted by a fitness blogger as of April.
Length	The character length of the bulletin content on a fitness blogger's homepage.
Time	The number of months since a fitness blogger first contributed a fitness video on the OFC to the data collection day.
Identity authentication (Auth)	Dummy variable to show whether a fitness blogger has identity authentication on his/her homepage. Auth = 1 if there is identification authentication, but zero otherwise.

### 4.4 Model construction

By collecting and analyzing the real data from a popular OFC in China, this research investigated how online information of fitness bloggers affects the spread breadth and recognition of fitness knowledge generated by the fitness bloggers in online communities. To test the above hypothesis, this research established following empirical model we used STATA software to employ statistical analysis and model estimation.

$$\begin{array}{rl} SB_{i}= & \alpha_{0}+\alpha_{1}CPA_{i}+\alpha_{2}Rep_{i}+\alpha_{3}Gender_{i}+\alpha_{4}Gender_{i}\times CPA_{i}\\ & +\alpha_{5}Gender_{i}\times Rep_{i}+\alpha_{6}CQ_{i}+\alpha_{7}NumVedio_{i}\\ & +\alpha_{8}Length_{i}+\alpha_{9}Time_{i}+\alpha_{10}Auth_{i}+\varepsilon_{b,i} \end{array}$$ (1)

$$\begin{array}{rl} SR_{i}= & \beta_{0}+\beta_{1}CPA_{i}+\beta_{2}Rep_{i}+\beta_{3}Gender_{i}+\beta_{4}Gender_{i}\times CPA_{i}\\ & +\beta_{5}Gender_{i}\times Rep_{i}+\beta_{6}CQ_{i}+\beta_{7}NumVedio_{i}\\ & +\beta_{8}Length_{i}+\beta_{9}Time_{i}+\beta_{10}Auth_{i}+\varepsilon_{r,i} \end{array}$$ (2)

where *i* indicates fitness bloggers. $\alpha_{0}$ and $\beta_{0}$ are constant terms. $\alpha_{j}(\;j=1,2,\ldots ,10)$ is the regression coefficients indicated the variables' effect on spread breadth (*SB*). $\beta_{j}(\;j=1,2,\ldots 10)$ is the regression coefficients indicated the variables' effect on spread recognition (*SR*). This research employs Ordinary Least Squares (OLS) to estimate the above-constructed model equation.

## 5 Results

### 5.1 Descriptive statistics and regression diagnostics

Table 2 shows the descriptive statistics and Pearson correlation coefficients of the variables. The results show that independent variables (CPA, Rep) correlate significantly with dependent variables (SB, SR). The independent and control variables show low correlations. To verify the assumptions underlying the OLS regression, we further conducted diagnostic tests on the two main models. First, to examine the linearity assumption, we re-estimated the SB model using a log-transformed reputation variable as a robustness check; the results showed consistent signs and significance levels for all independent variables, confirming the appropriateness of the linear specification. Second, all Variance Inflation Factors were below 2.0 (mean VIF = 1.34), well below the threshold of 10, indicating no multicollinearity concerns. Third, the Breusch-Pagan test (SB: *χ*^2^ = 3,394.08, *p* < 0.001; SR: *χ*^2^ = 4,532.44, *p* < 0.001) and White's test (SB: *χ*^2^ = 460.33, *p* < 0.001; SR: *χ*^2^ = 343.69, *p* < 0.001) both rejected the null of homoscedasticity; therefore, robust standard errors were employed in all regression models. Fourth, as this study uses cross-sectional data with each blogger observed only once, the assumption of independence of errors is inherently satisfied. Finally, the Shapiro–Francia test (SB: *W*' = 0.40, *p* < 0.001; SR: *W*' = 0.27, *p* < 0.001) indicated non-normal residuals; however, given the large sample size (*N* = 1,561), the OLS estimators remain asymptotically normal based on the Central Limit Theorem, ensuring the validity of statistical inference.

**Table 2:** Descriptive statistics and correlations of variables.

Variables	Min	Max	Mean	Std.Dev.	1	2	3	4	5	6	7	8	9	10
1.SB	0	16,270,000	321,268	11,74,000	1									
2.SR	−3	15,68,000	14,940	79,652	0.879***	1								
3.CPA	0	387	6.89	19.36	0.185***	0.141***	1							
4.Rep	1	10,180,000	83,863	3,88,077	0.432***	0.225***	−0.004	1						
5.NumCom	0	4,80,340	11,777	29,601	0.523***	0.321***	0.067***	0.601***	1					
6.NumVideo	0	15,000	269.7	722.7	0.073***	0.030	0.496***	0.033	0.107***	1				
7.Length	0	67	27.32	18.78	0.096***	0.090***	0.048*	0.057**	0.102***	−0.005	1			
8.Time	0	147	39.88	20.78	−0.029	−0.042	−0.073***	0.095***	0.216***	0.067***	0.029	1		
9.Auth	0	1	0.147	0.355	0.257***	0.169***	−0.020	0.355***	0.468***	0.017	0.117***	0.248***	1	
10.Gender	0	1	0.569	0.495	0.083***	0.099***	0.038	−0.011	0.103***	−0.034	0.079***	−0.002	0.026	1

* *p* < 0.10.

** *p* < 0.05.

*** *p* < 0.01.

### 5.2 Empirical results

Table 3 presents the ordinary least squares (OLS) estimation results for the specified model. Table 3 presents hierarchical regression results for both models: columns (1)–(3) show the impact on SB (Equation 1), while columns (4)–(6) display the impact on SR (Equation 2). The baseline models (columns 1 and 4) include control variables (Numvideo, Length, Time, Auth, and NumCom) and moderator variables (Gender), their adjusted $R^{2}$ is 0.296 and 0.121, respectively. Columns 2 and 5 introduce independent variables (CPA and Rep), their adjusted $R^{2}$ increases to 0.338 and 0.136, and the models pass the *F*-test. Interaction terms are added in columns 3 and 6, with adjusted $R^{2}$ is 0.357 and 0.176.

**Table 3:** Estimation results.

Model	Impact on SB			Impact on SR		
Variables	(1)	(2)	(3)	(4)	(5)	(6)
NUMVIDEO	45.260 (1.51)	−87.018 (−1.32)	−55.924 (−1.00)	0.669 (0.57)	−7.055* (−1.78)	−4.920 (−1.62)
Length	2,510.716* (1.79)	2,057.677 (1.56)	2,070.330 (1.58)	219.066** (2.01)	190.756* (1.84)	196.275* (1.93)
Time	−8,839.096*** (−6.32)	−7,196.449*** (−5.37)	−6,553.405*** (−5.11)	−468.640*** (−4.60)	−397.218*** (−4.14)	−337.103*** (−3.82)
Auth	1,43,943.291 (1.10)	96,543.023 (0.77)	28,083.027 (0.23)	9,948.147 (1.06)	9,598.296 (1.02)	2,017.028 (0.23)
NumCom	20.882*** (7.89)	16.319*** (5.20)	14.805*** (5.34)	0.846*** (4.01)	0.747*** (3.36)	0.595*** (3.06)
Gender	60,189.059 (1.45)	73,662.441* (1.83)	89,111.467** (2.08)	9,919.644*** (3.43)	9,479.272*** (3.38)	10,942.927*** (3.82)
CPA		10,524.921*** (3.40)	4,405.637** (2.21)		588.364*** (2.93)	173.763** (2.11)
Rep		0.567** (2.53)	0.446*** (2.78)		0.011 (0.95)	−0.004 (−0.41)
Gender × CPA			11,784.966** (2.47)			806.019** (2.45)
Gender × Rep			0.750* (1.90)			0.086*** (2.96)
Constant	2,91,573.265*** (4.89)	2,07,109.923*** (3.40)	2,41,492.981*** (4.28)	10,391.763*** (2.86)	6,887.731* (1.77)	9,810.373*** (2.69)
*N*	1,561	1,561	1,561	1,561	1,561	1,561
Adjusted *R*^2^	0.296	0.338	0.357	0.121	0.136	0.176
*F*	15.084	14.190	15.646	6.396	4.857	4.747
Prob > *F*	0.000	0.000	0.000	0.000	0.000	0.000

Robust standard errors were employed in all regression models. *t* statistics in parentheses.

* *p* < 0.10.

** *p* < 0.05.

*** *p* < 0.01.

In column (2), the regression coefficient of CPA is positive and significant $\left(\alpha_{1}=10,524.921,p<0.01\right)$, indicating that the content production ability of fitness bloggers has a positive influence on the spread breadth. The regression coefficient of Rep is positive and significant $\left(\alpha_{2}=0.567,p<0.05\right)$, indicating that the fitness bloggers’ reputation positively impacts the spread breadth. Therefore, the hypotheses H1(a) and H2(a) are supported. The content production ability and reputation of fitness bloggers have a positive influence on the spread breadth of the created fitness knowledge in online communities.

In column (3), the coefficient of Gender × CPA is significant $\left(\alpha_{4}=11,784.966,p<0.05\right)$, indicating that the influence of content production ability on spread breadth is heterogenous across different genders. If a fitness blogger is male, the content production ability has a greater impact on the spread breadth than that of a female fitness blogger. Thus, H3(a) is supported. The coefficient of Gender × Rep is not significant $\left(\alpha_{5}=0.750,p>0.05\right)$, which indicates that H4(a) is not supported.

In column (5), the regression coefficient of CPA is positive and significant $\left(\beta_{1}=588.364,p<0.01\right)$, indicating that the fitness bloggers' content production ability has a positive influence on the spread recognition. Therefore, hypotheses H1(b) is supported. The regression coefficient of reputation is not significant $\left(\beta_{2}=0.011,p>0.05\right)$, thus, H2(b) is not supported. In summary, fitness bloggers' content production ability has a positive influence on the spread recognition of the created fitness knowledge in online communities, wheras reputation does not.

In column (6), the coefficient of Gender × CPA is significant $\left(\beta_{4}=806.019,p<0.01\right)$, it shows that if a fitness blogger is male, the content production ability has a greater impact on the spread recognition than that of a female blogger. Therefore, the influence of fitness bloggers' content production ability on spread recognition is heterogeneous among different genders; thus H3(b) is supported. Additionally, Gender × Rep is significant $\left(\beta_{5}=0.086,p<0.01\right)$, suggesting a moderating effect that warrants attention. Nevertheless, since the main effect of Rep on spread recognition is not significant in column (5), H4(b) is not supported based on our primary model.

### 5.3 Robustness check

To test the robustness of the above results, we changed the dataset to test the robustness of the constructed model and estimation. This study used a 2-month increment to replace the original 1-month increment in the number of views and likes, and we also used OLS to estimate the regression coefficients again. The estimation results are shown in Table 4. The estimation results of independent variables and interaction terms demonstrate consistent signs and significance levels with Table 3 results, confirming the robustness of our results.

**Table 4:** Estimation results.

Model	Impact on SB		Impact on SR	
Variables	(1)	(2)	(3)	(4)
NumVideo	−161.093 (−1.28)	−103.212 (−0.96)	−13.018* (−1.80)	−8.966 (−1.62)
Length	4,134.782* (1.66)	4,192.661* (1.68)	333.173* (1.81)	343.492* (1.89)
Time	−15,615.472*** (−5.97)	−14,314.337*** (−5.80)	−866.650*** (−4.66)	−754.775*** (−4.46)
Auth	2,71,235.601 (1.10)	1,49,694.632 (0.62)	24,693.247 (1.36)	12,372.962 (0.76)
NumCom	33.706*** (5.31)	30.841*** (5.41)	1.567*** (3.70)	1.304*** (3.41)
Gender	1,43,922.726* (1.79)	1,72,306.007** (2.01)	17,456.634*** (3.30)	19,975.751*** (3.67)
CPA	10,420.412*** (3.62)	3,902.337** (2.24)	606.702*** (3.31)	155.218** (2.19)
Rep	0.950** (2.41)	0.745** (2.52)	0.009 (0.54)	−0.013 (−0.73)
Gender × CPA		13,045.485*** (3.14)		907.561*** (3.52)
Gender × Rep		1.320 (1.57)		0.138** (2.50)
Constant	4,47,783.870*** (3.95)	5,12,645.916*** (4.83)	17,688.651** (2.57)	22,969.646*** (3.54)
*N*	1,561	1,561	1,561	1,561
Adjusted *R*^2^	0.342	0.362	0.155	0.190
*F*	13.678	15.620	5.508	5.314
Prob > *F*	0.000	0.000	0.000	0.000

Robust standard errors were employed in all regression models. *t* statistics in parentheses.

* *p* < 0.10.

** *p* < 0.05.

*** *p* < 0.01.

## 6 Discussion and implications

### 6.1 Discussion

In this paper, we explore how the features of fitness bloggers influence the spread of fitness knowledge in OFCs. Specifically, we study the influence of fitness bloggers' content production ability and reputation on the spread effect of fitness knowledge. We used the spread breadth and spread recognition to measure the spread effect of fitness knowledge, then we took the increment of view numbers and the increment of the number of likes as proxy variables to measure spread breadth and recognition, respectively. We hypothesized that content production ability and reputation can all positively influence the spread breadth and recognition of fitness knowledge. In addition, we hypothesized that gender can moderate the relationship between the fitness bloggers' content production ability or reputation and the spread effect. Using empirical data from Bilibili.com, a leading fitness content platform, we built and tested an empirical model. The analysis results provided strong support for the majority of our hypotheses.

The results show that fitness bloggers' content production ability positively influences both spread dimensions. Bloggers with higher production ability maintain frequent updates, which not only attracts new viewers but also retains existing users, building a loyal audience base that generates sustained recognition over time.

Furthermore, reputation positively impacted spread breadth but not spread recognition. While reputation serves as a credibility signal that attracts viewers and extends content reach, users' recognition through likes depends more on the content's personal relevance and production consistency than on the blogger's reputational standing alone.

Additionally, this research finds that the influence of fitness bloggers' content production ability on spread breadth and recognition is heterogeneous among different genders. If a fitness blogger were male, the influence of content production ability on the spread breadth and spread recognition would be greater. Therefore, to expand the generated content's spread scope and enhance its spread effect, male fitness bloggers should improve their ability to track emerging fitness trends and technological developments, provide timely and practical information, improve update frequency, and continue to attract the users' attention and interests.

### 6.2 Theoretical implications

This research has following theoretical significance in four aspects. First, in internet fitness, there is limited literature focused on fitness knowledge sharing and spreading in OFCs. Existing research on the influence of fitness bloggers’ features on the spread effect is limited. Within the context of OFCs, this study summarized the fitness bloggers' features into two aspects: content production ability and reputation, and we demonstrated how these features impact the spread of fitness knowledge in online communities. Furthermore, this study explored the heterogeneous influence of fitness bloggers' features on spreading fitness knowledge among different genders in online communities. This study provided a new perspective on researching online fitness knowledge sharing and spreading.

Second, this study introduces a health economics lens to the study of online fitness knowledge dissemination. By conceptualizing fitness knowledge as a health-related good exchanged in a digital market, and bloggers' content production ability and reputation as quality signals that mitigate information asymmetry, this research extends the application of signaling theory to the domain of digital health communication. While Guo et al. (17) systematically reviewed signaling mechanisms in online health communities, empirical evidence on how specific supply-side signals differentially influence dissemination outcomes remains limited. Our study addresses this gap by demonstrating that content production ability serves as effective signals for both spread breadth and recognition, whereas reputation functions primarily as a breadth-enhancing signal. The differential effects of these signals on spread breadth vs. spread recognition provide nuanced empirical evidence on the efficiency of health information markets, contributing to the emerging literature at the intersection of health economics and digital health promotion.

Third, this study extended the theory of spread effects to research online fitness knowledge. Compared to previous research, this paper measures the spread of fitness knowledge using spread breadth and spread recognition. The study finds that the influence of fitness bloggers' features on the spread effects of fitness knowledge is inconsistent. The content production ability of fitness bloggers positively impacts the spread breadth and spread recognition of fitness knowledge. In contrast, the reputation of fitness bloggers has a positive influence on the spread breadth, but no significant influence on spread recognition. This research provides a detailed perspective that fitness bloggers with a high reputation are not necessarily recognized by their fans.

Fourth, it is the first time to use real data collected from online fitness community platforms to establish empirical regression models and study the spreading of fitness knowledge. The research methods of this study can provide new ideas for future research in the field of sports and fitness.

### 6.3 Practical implications for digital health promotion and resource allocation

Our findings have several implications for the efficient allocation of digital health promotion resources. Firstly, fitness bloggers seeking broader reach should build a strong reputation to attract larger audiences, while sustained high-quality content production is essential for both expanding reach and gaining user recognition. Notably, good reputation alone does not guarantee recognition—loyalty and endorsement are earned through high-quality output. For male bloggers in particular, strong content production ability is especially critical for attracting users and disseminating fitness knowledge. Secondly, OFC platforms can optimize health information delivery by incentivizing high-quality content through traffic recommendations and priority exposure, introducing content challenges to sustain bloggers' creative vitality, and enhancing interactive features to strengthen blogger-user connections and retention. These measures can improve the cost-effectiveness of online health communication and foster a more efficient fitness information ecosystem.

### 6.4 Limitations and future research

This research has some limitations, so we propose some suggestions for future research. First, analyzed data was collected from only one OFC (Bilibili's Aerobics section) in China. Although it is one of the largest OFCs in China, the results may be influenced to some extent by the country's cultural and platform context. Our findings need to be validated on other OFCs. Future research could investigate this topic across cultures to better understand the impact of fitness knowledge spread from a global perspective. Second, this research used cross-sectional data to test the hypotheses. The dataset lacked longitudinal tracking of fitness bloggers' online information evolution, which limits causal identification. Omitted variable bias is also a concern inherent in observational studies. Future research could employ longitudinal panel data with fixed effects, instrumental variable approaches, or quasi-experimental designs to strengthen causal inference. Third, the spread effect in this study was measured by spread breadth and spread recognition, primarily reflecting increases in views and likes. Future research might incorporate more comprehensive measures of the spread effect, including increments in follower numbers and shares, to provide a more holistic assessment of the online spread effect of fitness knowledge. Fourth, apart from the influence of CPA and Rep on the dissemination effect, the characteristics of fitness knowledge itself, such as the topic and quality of the content, may also affect the dissemination of fitness knowledge. In the future, a more detailed analysis can be conducted by collecting the fitness videos of the bloggers. Fifth, due to platform data constraints, only gender was examined as a moderating variable. Future research with access to richer demographic data should investigate whether age and socioeconomic background moderate the signaling effects identified here.

## 7 Conclusion

Online fitness knowledge is an important supplement to Internet fitness services, and users can find fitness bloggers through OFCs to further understand fitness knowledge. This mechanism can use existing internet resources and bring great convenience to users. However, the research associated with fitness knowledge spread is limited. To address this research gap, we constructed a theoretical model and conducted an empirical analysis to explore how the content production ability and reputation of fitness bloggers impact the spread of fitness knowledge. We also investigated the above features' heterogeneous influence among different genders. The analysis results provided strong support for the majority of our hypotheses. By framing fitness knowledge as a health-related good exchanged in a digital market and bloggers' attributes as quality signals that mitigate information asymmetry, this study offers an empirical assessment of the supply-side factors shaping the efficiency of health information dissemination in online communities.

To sum up, by identifying the differential effects of content production ability and reputation on the spread breadth and spread recognition of fitness knowledge, this paper contributes to understanding how health-related information is produced, signaled, and consumed in digital communities. These findings provide actionable insights for improving the allocative efficiency of digital health promotion resources and for designing more cost-effective, equity-oriented online health communication strategies.

## Data Availability

The raw data supporting the conclusions of this article will be made available by the authors, without undue reservation.
